# A Comparative Study on the Effect of Task Specific Training on Right Versus Left Chronic Stroke Patients

**DOI:** 10.3390/ijerph17217950

**Published:** 2020-10-29

**Authors:** Reem M. Alwhaibi, Noha F. Mahmoud, Hoda M. Zakaria, Wanees M. Badawy, Mahmoud Y. Elzanaty, Walaa M. Ragab, Maher S. Benjadid, Nisreen N. Al Awaji, Hager R. Elserougy

**Affiliations:** 1 Rehabilitation Sciences Department, Health and Rehabilitation Sciences College, Princess Nourah bint Abdulrahmaan University, Riyadh 11671, Saudi Arabia; rmalwhaibi@pnu.edu.sa (R.M.A.); NFMahmoud@pnu.edu.sa (N.F.M.); 2 Department of Neuromuscular Disorders and its Surgery, Faculty of Physical Therapy, Cairo University, Cairo 12613, Egypt; dr_hodazakaria@yahoo.com (H.M.Z.); wanees.alamir@pt.cu.edu.eg (W.M.B.); mahmoud_pt@yahoo.com (M.Y.E.); ragab_walla@yahoo.com (W.M.R.); 3 Department of Physical Therapy for Neurology and Neurosurgery, Faculty of Physical Therapy, Delta University for Science and Technology, Dakahlia 11152, Egypt; 4 Department of Neuromuscular Disorders and its Surgery, Faculty of Physical Therapy, Deraya University, New Menya 11159, Egypt; 5 Department of Physical Therapy, Faculty of Medical Rehabilitation Sciences, Taibah University, Medina 42353, Saudi Arabia; 6 Rehabilitation Medicine Department, Prince Sultan Military Medical School, Riyadh 11159, Saudi Arabia; maljadidd@psmmc.med.sa; 7 Health Communication Sciences Department, College of Health and Rehabilitation Sciences College, Princess Nourah Bint Abdulrahman University, Riyadh 11671, Saudi Arabia; NNAlAwaji@pnu.edu.sa; 8 Department of Neuromuscular Disorders and its Surgery, Faculty of Physical Therapy, Misr University for Science and Technology, Giza 77, Egypt

**Keywords:** stroke, motor impairment, task specific training, motor brain area, sensory brain area, quantitative electroencephalogram

## Abstract

Functional impairment of the upper limb (UL) after stroke is a great problem. Finding methods that can improve UL function after stroke is a major concern to all medical service providers. This study was intended to compare the effect of upper limb task specific training (TST) on brain excitability of the affected hemisphere and motor function improvements in patients with left and right stroke. Forty male patients with mild impairment of UL functions were divided into two equal groups; G1 consisted of patients with left hemisphere affection (right side stroke) while G2 consisted of patients with right hemisphere affection (left side stroke). All patients received TST for the affected UL for one hour, three sessions per week for six consecutive weeks. Evaluation was performed twice, pre-, and post-treatment. Outcome measures used were Wolf Motor Function Test (WMFT) and Box and Block Test (BBT) as measures of UL motor function and Quantitative Electroencephalogram (QEEG) of motor and sensory areas of the affected hemisphere as a measure of brain reorganization post-stroke. Both groups showed improvement in motor function of the affected UL measured by WMFT and BBT with reported significant difference between them. G1 showed greater improvement in motor function of the affected UL post-treatment compared to G2. Additionally, there was a significant increase in peak frequency of motor and sensory areas with higher and significant excitability in G1 only. These findings imply that brain reorganization in the left hemisphere responded more to TST compared to the right hemisphere. Based on findings of the current study, we can recommend adding TST to the physical therapy program in stroke patients with left hemisphere lesions.

## 1. Introduction

Stroke is considered one of the main causes of disability in adults. Loss of upper limb (UL) function is a direct result of stroke. Although about 83% of stroke patients walk again, approximately 65% of them suffer from long-standing limitations in UL function [1]. Common UL impairments after stroke include paresis, abnormal muscle tone, and/or changes in somatosensation. These impairments are caused by direct damage to the primary motor and somatosensory cortex, secondary sensorimotor cortical areas, subcortical structures, and/or the corticospinal tract [2]. Due to the residual disability caused by stroke, there is an increased incidence of mortality. This is why selecting proper treatment strategies that help overcome the adverse effects of stroke disability is crucial [3]. Patients with stroke experience loss of motor control. Currently, treatment of motor deficits in stroke adopt the concept of motor learning; or more accurately, the motor relearning approach [4]. An important principle of motor relearning is specificity of practice or in other words task-specific training (TST) [5]. TST involves integrating tasks that are commonly used in various activities of daily living (ADL) (e.g., holding a cup) into the rehabilitation program [6]. Movements performed consist of motor tasks that focus on an improvement in performance through goal-oriented practice and repetition [7] with the aim of acquiring functional skills. It is well recognized that any movement is the result of interaction between the subject, the task, and the environment, where the task takes place [8,9].

Various terms are used to describe this treatment approach. For example, TST is used interchangeably with task-oriented training [8], task-related training, or repetitive task training [10]. When designing a TST program, it is recommended that the tasks are challenging, progress from easy to hard, are based on patient abilities, are practiced within different contexts and environments, aim toward mastering the whole task, include different types of feedback, and involve active participation of the individual [7].

Patient response to TST might be variable and may depend on whether the patient has right or left side hemisphere lesion. TST displays great changes in response and recovery in patients with stroke [11,12]. It is expected that continuous training compels brain reorganization and increases UL functional performance [13,14].

There is evidence that TST affects neural plasticity in stroke patients [15,16]. A systematic review and meta-analysis that involved transcranial magnetic stimulation (TMS) and functional magnetic resonance imaging (fMRI) reported improvements in neural plasticity of the sensorimotor cortex, which are reflected in improvements in function of the affected UL after using TST [16,17].

Quantitative electroencephalography (QEEG) is a neurophysiological technique that measures cortical activity and brain waves. It can determine the effect of various treatment approaches used for stroke patients [18]. This is why QEEG can help in detecting which treatment modality results in greater plasticity and functional improvement. QEEG has four frequency bands: delta, theta, alpha, and beta with frequency ranges of (1–4 Hz), (4–8 Hz), (8–12 Hz), and (12–30 Hz), respectively [19,20].

To our knowledge, no studies have been carried out to investigate and analyze the effects of TST on motor and sensory areas of the brain in stroke patients with right and left side hemispheric lesions. This is why the aim of the current study was to compare the effect of UL TST on brain excitability of the affected hemisphere and motor function improvements in patients with left and right stroke.

## 2. Materials and Methods

### 2.1. Participants

The current randomized controlled clinical trial study was approved by the ethical committee of the Faculty of Physical Therapy, Cairo University, Egypt (P.T.REC/012/002153). Fifty seven male stroke patients (*n* = 57) were recruited from the Outpatient Clinic of the Faculty of Physical Therapy, Cairo University and evaluated for eligibility, eleven (*n* = 11) were excluded for not meeting the criteria and forty-six (*n* = 46) were randomly assigned to two equal groups (G1 and G2). Six patients (*n* = 6) refused to provide consent and dropped out of the study. Patients were included in the study if age ranged from 47–64, diagnosed with first ever ischemic/hemorrhagic stroke resulting in hemiparesis; confirmed by Magnetic Resonance Imaging (MRI) and the treating neurologist, stroke duration ranged from 6–24 months, medically stable (based on treating neurologist recommendations), scored >24 on the Mini-Mental State Examination (MMSE); joints of the affected UL scored grade 1 on the Modified Ashworth Scale (MAS); mild impairment on Fugl Meyer Scale-Upper Extremity (FMA-UE), and finally if the affected UL scored 2 for tactile sensation and stereognosis 3 for kinesthetic sense on the Nottingham Sensory Assessment (NSA) scale. Patients were excluded from the study if they suffered from any of the following: cardiac arrhythmia, uncontrolled hypertension, obstructive pulmonary disease, or any previous orthopedic or neurological problem in the affected UL such as polyneuritis other than stroke. A proper explanation of the treatment protocol was given, and a consent form was signed by all participants.

### 2.2. Randomization

Having given informed consent, all stroke patients were randomly assigned by a random number computer-generator, into two equal groups (G1 and G2). G1 consisted of patients with left hemisphere lesion and G2 consisted of patients with right hemisphere lesion. All the patients were right-handed, determined by the Edinburgh handedness inventory, which means the dominant hemisphere was left. Both groups received the same program of treatment, TST.

### 2.3. Sample Size

Calculation of means and standard deviation were obtained from a pilot study that included 10 patients with stroke who underwent the same interventions of the current study. Sample size calculation was performed prior to the study using G*POWER statistical software (version 3.1.9.2; Franz Faul, Universitat Kiel, Germany) and revealed that the required sample size for the current study was 20 patients per group. Calculations were made using α = 0.05, β = 0.2 and effect size = 0.91 and allocation ratio N2/N1 = 1. A total of 46 were recruited to compensate for possible dropouts from the study (16% drop rate *n* = 6).

### 2.4. Clinical Examination

All patients were evaluated pre- and post-treatment using the Wolf Motor Function Test (WMFT), Box and Block Test (BBT), and Quantitative Electroencephalogram (QEEG). The WMFT was used to measure functional ability of the affected UL. The BBT was used as a measure of gross manual dexterity of the affected UL. QEEG was used to measure the brain activity of motor areas C3/C4 and sensory areas P3/ P4. Demographic data were collected pre-treatment from each patient. All evaluation procedures and data collection were performed at baseline (W0) and after six weeks of intervention (W7).

#### 2.4.1. Wolf Motor Function Test (WMFT)

The WMFT examines UL functional ability, dexterity, and strength using two strength measurements and a series of 15 functional tasks that progress from simple movements in proximal joints to complex movements in distal joints. Each of the 15 tasks is timed, up to a maximum of 120 s for completion [21]. The WMFT’s timed tasks measure the output speed, thus making it easy to identify improvements in functional performance of the affected UL. The WMFT was found to be a valid and reliable measure of UL function in mild [22,23] to moderately affected [24,25] patients with high test-retest and inter-rater reliability [26].

In this study, the motor output and performance time sections of the WMFT were used to calculate the functional performance of the affected UL. Each patient was seated and asked to carry out every item of WMFT as quickly as possible with a time limit of 120 s [23].

#### 2.4.2. Box and Block Test (BBT)

The BBT evaluates unilateral gross manual dexterity. The test utilizes repeated movements that require pick-up to release movement [27]. This test has a test-retest reliability greater than 0.9 [28,29].

In this study, each patient was seated and a divided box containing 150 blocks was placed in front of them on a table. The patient was allowed a 15 s trial period. Before the test began, each patient was instructed to place their hands on the sides of the box. When testing began, each patient was instructed, with the affected hand, to grasp one block (2.54 cm^3^) at a time, by the tip of the index finger and thumb, transport the block over the partition, and release it into the opposite compartment, for a duration of one minute [30]. A stopwatch was used to document time. High scores suggest greater gross manual dexterity.

#### 2.4.3. Quantitative Electroencephalogram (QEEG)

QEEG [31] was used to record the pattern of brain waves of the affected hemisphere (whether right or left). The study was conducted on a digital EEG-EP Multifunction System 10–20 Channel (Wide Band) (EBNeuro, Florence, Italy/Mizar-Pc Peripheral System CE Version-B9800037800).

A clinical neurophysiologist performed the neurophysiological test while the patients laid comfortably on their backs with closed eyes to minimize artifacts from eye movements or any visual feedback. Data were recorded according to the International 10–20 system with Ag/AgCl electrodes, using a unipolar montage while the impedance was kept constant.

The recorded data were then transformed into a mapping program to perform spectral analysis. The parameter used in the current study was the peak frequency of alpha waves. The brain function and plasticity of motor areas C3/C4 and sensory areas P3P4 in the affected hemisphere were measured by QEEG for both groups. The odd numbers (C3–P3) were used as a subscript for points over the left affected hemisphere in G1, while the even numbers (C4–P4) over the right affected hemisphere were in G2.

### 2.5. Therapeutic Interventions

Patients in (G1 and G2) received three sessions per week for six consecutive weeks (total of 18 sessions). Session duration was one hour and consisted of TST. Patients in both groups were asked to practice the same exercises at home.

Selected TST exercises were based on Carr and Shepherd [13] and included six exercises as follows:

(a) simulating drinking water from a glass;
(b) lifting a glass of water to a level of 90° shoulder flexion with an extended elbow;
(c) moving 5 tennis balls from the table to a box;
(d) wiping the table with a towel with the elbow extended;
(e) moving a cone from a table to a shelf; and
(f) combing hair.

During each one-hour session of TST, all patients performed warm-up exercises for 10 minutes (min), then practiced the selected tasks for the remaining 50 min. During these 50 min of TST, a two-min rest period followed every 15 min of continuous practice. Before training, tasks were demonstrated for each patient with reference to their unaffected UL. Variables such as speed, distance, or/and resistance progressively increased in difficulty according to each patient’s ability. The treating physical therapist provided verbal, visual, or proprioceptive feedback and manually assisted patients to ensure they performed all the required tasks precisely [32,33].

### 2.6. Statistical Analysis

Paired and unpaired t-test were used to compare the peak frequency of alpha waves of motor areas C3/C4 and sensory areas P3 /P4 within and between both groups while Wilcoxon and Mann–Whitney tests were used to compare changes in scores of the WMFT within and between groups. Unpaired t-test and chi squared test were used in demographic data analysis. The level of significance was set at *p* ≤ 0.05

## 3. Results

**Figure 1:**
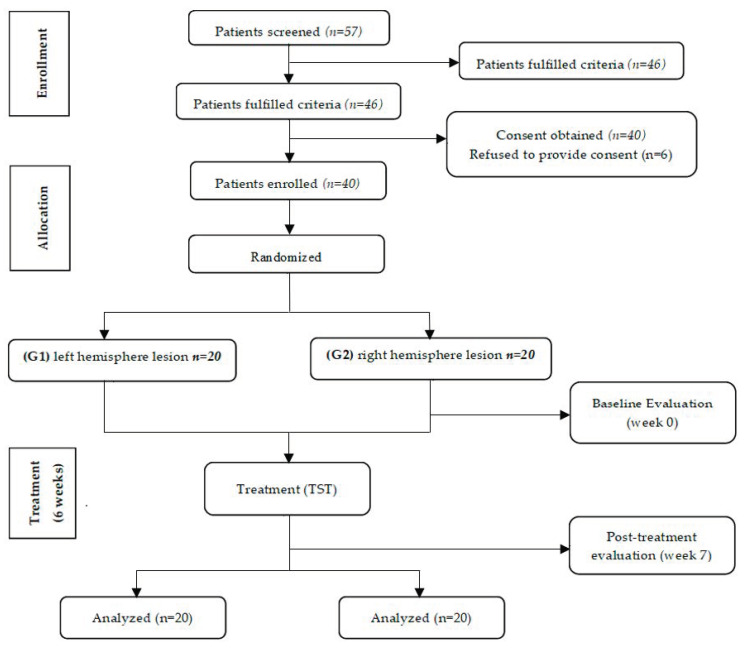
Flow diagram of the study.

A total of 57 stroke patients were screened for eligibility. Of these, 46 were eligible to participate in the study. Six patients refused to provide consent and 40 completed the study (Figure 1).

### 3.1. Demographic Data

The demographic characteristics of patients in both groups did not differ significantly (*p* > 0.05) (Table 1).

### 3.2. Clinical Scales

Data presented in Table 2 and Table 3 show that there was no significant difference between both groups, pre-treatment in scores of the WMFT and BBT of the affected UL, while post-treatment, there were significant differences within each group and between groups with a greater significant increase in scores post-treatment in group 1 compared to group 2 (*p* < 0.05).

### 3.3. Quantitative Electroencephalogram (QEEG)

Results in Table 4 showed no significant difference between both groups pre-treatment in the peak frequency of the alpha wave of motor areas (C3/C4). On the other hand, there were significant differences within the group 1 scores (*p* = 0.0005) and between both groups post-treatment in the peak frequency of the alpha wave being higher in group 1 (*p* = 0.001).

Data presented in Table 5 showed no significant difference between both groups, pre-treatment in peak frequency of the alpha wave of sensory areas (P3/P4), or within group 2 when comparing pre-treatment scores with post-treatment ones. Still, there was a significant difference within group 1 scores (*p* = 0.005) and between groups, post-treatment in peak frequency of the alpha wave of sensory area (P3/P4), being higher in group 1 (*p* = 0.011).

## 4. Discussion

In this study, we found that TST produced significant improvements in the functional ability of the affected UL, which were similar in patients with left hemisphere lesion (group 1) and right hemisphere lesion (group2), and confirmed by post-treatment scores of WMFT and BBT. On the other hand, results of QEEG showed more significant improvement in the brain activity of motor and sensory areas in patients with left hemisphere affection, compared to right hemisphere affection. Results of QEEG agreed with those obtained by the WMFT and BBT, but in group 1 only.

This discrepancy in response to TST, observed in patients with right and left hemisphere might be attributed to language acquisition, one of the most lateralized functions of the human brain. Language dominance in the left hemisphere has been consistently accepted in clinical and experimental settings and is one of the main axioms of neurology and neuroscience. TST depends on verbal command in functional forms, that is why, in the current study, patients with left hemisphere lesion responded to TST more than those with right hemisphere lesion as patients in our study had no problem with speech [34].

TST is based on the concept that the best way to learn a particular task is through continuous practice. The planned activities of TST were found to be realistic, purposeful, and important for the functionality of patients [35,36]. This explains why there was an increase in the peak frequency of the alpha wave of motor areas in both groups because this type of exercise enhances motor learning. On the other hand, the difference between both groups in response might be attributed to hemisphere involvement, as in the current study, the left hemisphere responded more to functional tasks compared to the right hemisphere [37].

The improvement in the sensory areas might attribute to feedback during task performance. Feedback enhances proper motor performance, which improves patient motivation. Additionally, feedback on misperformance is even more successful in promoting the development of skills. Hence during this study, we instructed patients on how to perform functional movement and then corrected it in the following session. Skilled performance feedback is acquired through intrinsic and extrinsic feedback mechanisms while practicing a task [38]. Task-intrinsic feedback was provided in this study by visual and proprioceptive feedback from the patient performing the task. In our study, the task-extrinsic or enhanced feedback included verbal encouragement. Additionally, the results in our study agreed with Fritsch et al. (2010) [39], who mentioned that when the task is assumed to be implicitly mastered (i.e., without acquiring clear knowledge of the sequence order), a significant increase in the activity of sensorimotor cortex, left motor and premotor cortices can be observed, along with supplementary motor areas along with increased blood flow. This might explain why sensory area excitability in the left hemisphere was greater than the right hemisphere.

As the left hemisphere is dominant for speech and verbal command, sensory augmentation was greater in group 1, along with motor excitability due to links and projections present between motor and sensory areas. Additionally, brain damage impairs stroke of intrinsic feedback systems, and they have to rely more on extrinsic feedback for motor learning. Extrinsic feedback may be classified as results knowledge (KR) or performance knowledge (KP), summary feedback (overview of previous test results) or average feedback (average of previous test results), bandwidth feedback, qualitative or quantitative feedback, and may be given at the same time or at the end of the task performance (terminal feedback) [38]. The left hemisphere is specialized in predictive control, the ability to plan and organize motor behavior efficiently, likely by optimizing certain cost functions. On the other hand, right hemisphere circuits seem important for updating ongoing behavior and stopping at a target position by modulating mechanisms of sensorimotor stabilization such as reflexes [40,41].

The left-hemisphere focuses on movement preparation, resulting almost entirely from the use of “routine” activities such as reaching actions. The function of the right hemisphere might be to detect and respond to unforeseen environmental stimuli [42]. Improvement of movement seen in group 2 with right hemisphere lesion is thought to represent improvements in predictive control intensified during TST.

According to Abdullahi (2018) [43], a linear relationship exists between the quantity of repetitions and UL motor function recovery. Tasks should be repeated at high rates without causing pain or fatigue, in order to achieve significant improvements in motor function and utilizing the affected UL post stroke. Additionally, the effectiveness of interventions is also directly influenced by factors such as repetition, time, proper use of training, and motivation [44]. Despite high intensity and dose of TST having already been studied [45], additional studies are required to investigate optimal TST protocols required to improve UL motor control.

### Limitations

The outcome measures of UL functional activity used in the current study measured motor function only, while QEEG measured brain activity of both sensory and motor areas. The long effect of the TST program was not evaluated. Patient adherence to home program was not assured. Future studies are required to investigate the effect of practicing TST on different brain areas that contribute to motor performance.

## 5. Conclusions

Recent studies have not investigated the effects of TST on right and left motor and sensory areas and how that reflects on the function of the affected UL. Our results showed that brain activity in the left hemisphere compared to the right hemisphere was better, implying higher brain reorganization after TST and thus better brain plasticity. Additionally, UL motor function measured by WMFT and BBT improved more in patients with left side brain lesion compared to right. Accordingly, it is recommended that TST is added to the physical therapy program of in patients with right side hemiparesis (left hemisphere brain injury).

## Figures and Tables

**Table 1:** The mean values of demographic data in both groups.

Demographic Data	G1 (*n* = 20)	G2 (*n* = 20)	*p* Value
Age (years)	55.8 ± 5.7	56.6 ± 4.9	0.584 ^a^
Type (Infarction/Hemorrhage)	12/8	13/7	0.652 ^b^
Height (cm)	166.1 ±7.21	164.7 ± 8.34	0.784 ^a^
Weight (kg)	65.2 ± 9.42	67.3 ± 11.31	0.651 ^a^
Duration of stroke (month)	23.22 ± 2.09	22.11± 3.07	0.949 ^a^

^a^ Unpaired t-test, ^b^ Chi-squared test.

**Table 2:** The mean values of Wolf Motor Function Test (WMFT) of the affected UL in both groups.

WMFT	G1	G2	*p* Value
Pre Test	24.29 ± 9.1	21.86 ± 8.3	0.45 ^b^
Post Test	55.86 ± 9.87	46.14 ± 12.8	0.04b *
*p* Value	0.0001 ^a,^*	0.0001 ^a,^*	-

^a^ Wilcoxon test; ^b^ Mann–Whitney test; * Significant at *p* < 0.05.

**Table 3:** The mean values of Box and Block Test (BBT) of affected UL in both groups.

BBT	G1	G2	*p* Value
Pre Test	22.9 ± 12.1	23.75 ± 14.4	0.62 ^b^
Post Test	31.45 ± 10.47	29.96 ± 10.17	0.005 ^b,^*
*p* Value	0.001 ^a,^*	0.002 ^a,^*	-

^a^ Wilcoxon test; ^b^ Mann–Whitney test; * Significant at *p* < 0.05.

**Table 4:** The mean values of peak frequency of alpha wave of motor areas (C3/C4) of the affected hemisphere in both groups.

QEEG (C3/C4)	G1	G2	*p* Value
Pre Test	8.501 ± 0.1	8.326± 0.13	0.394 ^b^
Post Test	9.443 ± 0.4	8.298 ± 0.03	0.001 ^b,^*
*p* Value	0.0005 ^a,^*	0.886 ^a^	-

^a^ Paired t-test; ^b^ Un-paired t-test; * Significant at *p* < 0.05.

**Table 5:** The mean values of peak frequency of the alpha wave of sensory areas (P3/P4) of the affected hemisphere in (G1) and (G2).

QEEG (P3/P4)	G1	G2	*p* Value
Pre Test	8.96 ± 0.63	8.876 ± 0.4	0.832 ^b^
Post Test	9.97 ± 0.86	8.652 ± 0.5	0.011 ^b,^*
*p* Value	0.005 ^a,^*	0.665 ^a^	-

^a^ Paired t-test; ^b^ Un-paired t-test; * Significant at *p* < 0.05.
